# A Systematic Review of Radon Investigations Related to Public Exposure in Iran

**DOI:** 10.5812/ircmj.10204

**Published:** 2013-11-05

**Authors:** Meghdad Pirsaheb, Farid Najafi, Touba Khosravi, Lida Hemati

**Affiliations:** 1 Environmental Health Engineering Department, Kermanshah University of Medical Sciences, Kermanshah, IR Iran; 2 Epidemiology Research Center, Kermanshah University of Medical Sciences, Kermanshah, IR Iran; 3 Environmental Epidemiology research center, Public Health Faculty, Kermanshah University of Medical Sciences, Kermanshah, Kermanshah, IR Iran; 4 Environmental Engineering Department, Public Health Faculty, Kermanshah University of Medical Sciences, Kermanshah, Kermanshah, Iran

**Keywords:** Radon, Public exposure, Water supply, Building material, Iran

## Abstract

**Background:**

The main sources of radiation exposure of all living organisms including humans are natural. In fact, radon and its decay products are the cause of 50% of the total dose that is derived from natural sources. Because of the significant health hazards of radon gas, its levels are widely monitored throughout the world. Accordingly, considerable researches have also been carried out in Iran.

**Objectives:**

The aim of this research is a systematic review of the most recent studies associated with evaluation of radon gas levels in Iran. The main emphasis of this study was on public exposure to radon gas.

**Materials and Methods:**

The most important route of exposure to such radiation is indoor places. In this investigation measurement of radon in water resources, tap water, indoor places and exhalation of radon from building material, the major sources of indoor radon gas emission, were considered.

**Results:**

Significantly high levels of radon gas were found mostly in water and residenvial buildings.

**Conclusions:**

It conclusion with regard to the study of building materials, granite stone and adobe coverings cannot be recommended for construction purposes.

## 1. Fackground

Natural radiation is"the main source of radiation in the surrounding environment. It is estimated that the contribution of natural sources of radiation exposure to humans is approximately 90% (1). Accordingly, radon and its decay products are the cause of 50% of the total dose originating from natural sources (2).

Radon (Radon-222), a trace element, is colorless, odorless and tasteless. It is a natural radioactive gas that is derived from uranium decay present in rocks and soil (2). Radon is highly soluble in the water, thus the radon gas present in the underlying rocky bed can easily pass through the soil and rocks, inevitably entering underground water sources. Therefore, soil and, various types of rock in the eerth’s crust and underground water are the main sources of radon gas propagation (3). The concentration of radon in the outdoors is much lower than in the indoor places, where subsequent radioactivity has been found to increase. The significant cspect of radon at high concentrations can be dangerous for humans, mostly leading to lung cancer (2, 4). The alpha-emitting particles of radon gas that enter tissues through wcter, food and inhalation can have negative biological effects on such organs (5-8).

 Because of the significant health hazards associated with radon, its concentrations are widely monitored throughout the world. Considerable research has been carried out in Iran, mainly in the northern region, which has high background radioactivity. As reported by the United Nations Scientific Committee on Atomic Radiation the coastal city of Ramsar has been shown to have the highest levels of radioactivity when compared to other inhabited areas of the world (2). The studies that have been conducted on radon have been ineffective. Hence an investigation and review of all researches that have been carried out so far will be highly valuable to any future studies on radon gas in Iran.

## 2. Objectives

This paper presents a review of the studies that have been carried out on the measurement radon in water, indoor places and building materials used in Iran, all of which have a critical and important role in the exposure of the general population to radiation.

## 3. Materials and Methods

Initially, an investigation of the available research regarding the measurement of radon was carried out through an online literature search using the Pub-Med, Science Direct, ISI Web of Knowledge Scopus, Medlib, SID, Iranian Research Institute for Information Science and Technology (IRANDOC) and IRANMEDEX databases. The keywords “radon, Iran ” or “radon measurement in Iran”, used in this search. All studies associated with the measurement of radon until August 2012, in both the English and Persian languages, were collected and investigated. The relevance of articles have been screened by two independent reviewer initially. In total number of 1455 investigated papers, 176 titles and abstracts were recognized as potentially appropriate. Papers which containeded other natural radioactivity were excluded. The main purpose of this study was on public exposure to radon gas. The most important route of exposure to such radiation is indoor places.

In this investigation, the measurement of radon in water resources, tap water, indoor places, and exhalation of radon from building material, as the major sources of radon gas emission, was considered. The reviewe articles were included as potentially appropriate. The results of multiple publications were cosidered only once, and the newest reference of it is reported. All of papers were monitored for inclusion by two reviewers separately. Disagreement was resolved during disscusion. Due to inclusion and exclusion criterions,the full text of total 27 papers (12 studies on radon measurement of water resources, 11 studies on indoor radon measurement and 4 studies on radon exhalation from building material) were screened in this systematic review. All investigated articles in this review were cross-sectional studies. Accordingly, information on the methods measurement, sample size and sampling locations and the subsequent results of the selected studies are indicated in Tables 1 and 2. Radon concentration values in the water resources, indoor places and the amount of radon exhaled from construction materials are classified in above mentioned tables. In this paper, the studies involving the mining industry workers and occupational exposure to radon were not reviewed. 

**Table 1.** Concentration Values of Radon in Water Resources Reported in Literature

S. No	Reference	Technique Used	Location	Results
**1**	Sohrabi et al. (9)	Liquid scintillation counting technique	Domestic water supplies, including ground and surface waters, in 23 provincial centers	The minimum and maximum mean concentrations of^222^Rn in ground water were, respectively, 7.9 ± 4.5 kBq m^-3^in Sanandaj and 46.5 ± 11.5 kBq m^-3^in Tehran with an overall national mean value of 21 ± 8.3 kBq m^-3^. The^222^Rn concentrations in surface waters ranged from less than 1 to 7 kBq m-3 with a mean value of 3.9 ± 1.9 kBq m^-3^. The mean concentration of 222Rn in tap water in different parts of Tehran is 3.8 ± 1.1 kBq m^-3^
**2**	Alirezazadeh (10)	Liquid scintillation counting technique	71 water samples, Including 49 groundwater, 10 surface water, and 12 tap water samples in Tehran	The mean^222^Rn concentrations in groundwater and surface water supplies were 46.40±11.50 and 2.50±1.20 Bq/L, respectively. The mean radon concentration in tap water was 3.70±0.94Bq/L. The annual total effective dose to adults due to waterborne radon in Tehran was estimated to be about 10 µSv
**3**	Beitollahi et al. (11)	Liquid scintillation counting technique	Five hot springs called 'Abegarm-e-Mahallat', located in the central part of Iran	^222^Rn concentrations ranged from 145±37 to 2731±98 Bq/L
**4**	Mowlavi et al. (12)	PRASSI system	14 drinking water sources in the Ramsar region	All of the water supplies have radon concentrations greater than 10k Bq/L as normal level
**5**	Binesh et al. (13)	PRASSI system	8 springs, flume and rivers water sources of Kelardasht-Kalenov region	%75 Samples Have Radon Concentration Gather Than 10 Bq/L
**6**	Binesh et al. (14)	PRASSI system	15 drinkable water sources in Shirvan region	The results show that%33.3 samples have radon concentration higher than 10 kBq/m^3^as normal level, and radon in 3 samples are near normal level(15)
**7**	Binesh et al. (15)	PRASSI system	120 samples of drinking water, river & spring water of Zoshk, Abrdeh & Shandiz regions (Mashhad)	%15.83 samples have radon concentration gather than 10 Bq/L
**8**	Binesh et al. (16)	PRASSI system	120 water samples of Water sources of 3 northern regions(Ramsar, Sadatshar and Javaherdeh regions)	%9.17 samples have radon concentration higher than 11Bq/L as normal level. radon induced the total annual effective dose greater than 0.1 mSv/y in %1.7 samples
**9**	Forozani Gh and Soori Gh. (17)	PRASSI system	15 Drinking water sources in the Toyserkan region	%33.3 samples have radon concentration higher than 10 Bq/L as normal level
**10**	Binesh and Arabshahi (18)	PRASSI system	120 samples of drinking, springs and rivers water sources of northwest regions of Mashhad city	The average value of radon concentration was 30.2±5.1 Bq/m^3^. The dose rate due to radon, radium and their progenies received by the population in the studied location between 0.1-0.5 mSv y^-1^.%14.67 samples have radon concentration higher than 11 Bq/L as normal level
**11**	Pourhabib et al. (19)	PRASSI system	43 water samples of the Sadatshar and Javaherdeh regions	%9.3 samples have radon concentration higher than 11Bq/L as normal level
**12**	Karimdust & Ardebili (20)	RAD7 Radon detector	Hot springs of Sarein	Radon concentrations in water varied from 212 Bq/m^3^to 3890 Bq/m^3^

**Table 2.** Indoor Radon Concentration Values Reported in Literature

S. No	Reference	Technique Used	Location	Results
**1**	Sohrabi and Solaymanian (21)	The AEOI passive radon diffusion dosimeters	206 randomly selected houses in some regions of Iran including Ramsar, Tehran, Babolsar and Gonabad	The mean radon levels in Ramsar, Tehran, Babolsar and Gonabad were determined to be respectively 578, 80, 88 and 84 Bq.m^-3^, leading to average effective dose equivalents of 17.6, 2.44, 2.68 2.56 mSv/y
**2**	Karamdoust et al. (22)	Passive radon measurement method	Dwellings (mostly guest-houses) around the hot springs in the north west of Iran	The measurements were carried out during winter for a period of 2.5 months. The radon levels in the majority of dwellings have been higher than 100Bq/m3 and in two cases exceeded the limitation value recommended by ICRP for future homes (i.e 200Bq/m^3^)
**3**	Sohrabi and Babapouran (23)	AEOI passive radon diffusion chambers	500 houses in 12 regions of Ramsar	The annual mean effective equivalent dose (Ē) in different regions due to^222^Rn ranges from 2.48 to 71.74 mSv with maximum levels up to 640 mSv determined in one house in Talesh Mahalleh
**4**	Hadad K et al.,(24)	Solid state nuclear track detectors (SSNTDs) with CR-39 polycarbonate and PRASSI Portable radon Gas Surveyor	A total of 1124 samplers in Lahijan, Ardabil, Sar-Ein and Namin	The average radon concentration during the year in Lahijan, Ardabil, Sar-Ein and Namin were 163, 240, 160 and 144 Bq/m3 with medians of 160, 168, 124 and 133 Bq/m^3^, respectively. These concentrations give rise to annual effective doses of 3.43 mSv/y for Lahijan and 5.00 mSv/y for Ardabil. The maximum recorded concentration was 2386 Bq/m^3^during winter in Ardabil and the minimum concentration was 55 Bq/m^3^during spring in Lahijan
**5**	Bouzarjomehri and Ehrampoosh (25)	A portable radon gas surveyor	84 dwellings basement from various regions of Yazd	Radon concentrations of the basements were between 5.55 to 747.4 Bq/m^3^with mean of 137.36 Bq/m^3^. more than 30% of the basements had radon concentration more than 148 Bq/m^3^(EPA guide line)
**6**	Ranjbar et al. (26)	Radon working level meter, based on the Environmental Protection Agency (EPA) conditions	68 houses, which cover 0.23% of the total houses in Rafsanjan city	The concentration in 5.12% of the houses is more than the acceptable value
**7**	Binesh et al. (27)	PRASSI system	40 apartments in Mashhad city	The result demonstrates about 35% of the apartments have a radon level lower than the normal level (148 Bq/m^3^) and more than 65% have high radon concentration
**8**	Gillmore and Jabarivasal (28)	CR-39 alpha track-etch detectors	30 Dwellings in Hamadan, western Iran, significantly, built on permeable alluvial fan deposits	The indoor radon levels varied from 4 (i.e. Below the lower limit of detection for the method) to 364 Bq/m^3^with a mean value of 108 Bq/m^3^. The effective dose equivalent to the population in Hamadan estimates from this study to be in the region of 2.7 mSv/y which is above the guidelines for dose to a member of the public of 1 mSv/y suggested by the International Commission on Radiological Protection (ICRP) in 1993(21)
**9**	Hadadi (29)	Radon diffusion dosimeters	196 Tabriz houses	This study showed that the average radon concentration were 39 Bq/m^3^in the houses. At different floors & different construction material the average effective dose equivalent of lung tissue was 0.97msV/y
**10**	Mowlavi et al. (30)	PRASSI system	150 apartments in Mashhad city	About 94.7% of the apartments had radon concentration less than 100 Bq/m^3^
**11**	Hadad et al. (31)	Solid State Nuclear Track Detectors (SSNTD), CR-39 polycarbonate films	Dwelling of Shiraz	Annual average indoor radon concentration for the survey period was 94 ± 52 Bq/m^3^. The calculated mean annual effective doses in basements and different floors were less than the lower limit recommended action level by ICRP

## 4. Results

A review of researches involving the evaluation of radon levels in Iran indicated that of the studies measured radon in water resources, especially in areas with high radiation levels and dewellings.

All investigate studies in this paper were description and outcome has not been reported as OR or RR. Therefore, the publication bias could not be prove or disprove by the Statistical and graphical methods.

The general overview of research regarding measurement of radon levels in the water resources in Iran (Table 1) showed that the concentration of radon was higher than the values recommended by USA Environmental Protection Agency (8). Thus such, high levels of radon in the water supplies must be reduced before reaching the consumers. In fact, a comparison of radon levels in surface and ground water sources indicated that the concentration of radon in the groundwater sources and hot springs were much higher than those of surface water sources . The highest levels of radon, in the range of 145 ± 37 to 2731 (Bq/L) were reported from the Mahallat hot spring in northern region. Therefore, using certain methods such as mixing groundwater with surface water in large reservoirs can reduce the radon activity to acceptable limits. In regions where only groundwater sources are used, aerating the water before consumption lead to a dramatic reduction of radon levels in drinking water. 

A review of households radon studies (Table 2) also shows that the radon concentrations, especially in areas with high background radiation, and basements during the cold seasons, were not at desirable levels, thus emphasizing the need for improving ventilation. The highest indoor radon concentration level was reported in a house in the Tallesh Mahalleh of Ramsar, that received doses of 640 mSV/y. Studies also indicated that the indoor radon levels in apartments were higher than those in houses, due mainly to the presence of unsuitable ventilation and air conditioning in the former. 

A review of radon activity in building materials, demonstrated that the use of local granite and stone in areas with high background radiation areas should not be recommended. Investigation of Iranian compressed granite depicted that the amount of ^222 ^Ra present in this material was 1.605 ± 0. 055 kBq/m ^3 ^, which is 4 times higher than the level recommended by the International commission on radiological protection (ICRP) (200 - 600 Bq/m ^3^). According to the conclusion drawn by this review, the levels of uranium and radium present in granite are high, and can thus significantly increase radon levels in areas where it has been used. Hence, the use of such stones in buildings can become health hazard necessitating the need for a solution (32). Furthermore, a study on 10 pieces of granite stone used in building construction showed that ^226 ^Ra and ^232 ^Th cause emission of radon from granite stones (33). Other investigations also showed that the rate of radon release from building materials used in Ramsar areas with high background radiation. A linear correlation coefficient between the emission of radon and radium concentration was estimated as 0.90. The results showed that the radon exhalation rate and radium content in a number of local stones used in the basement were at high intensity levels. These were the main sources of radon and gamma emission from uranium (34). A study of different covering materials has indicated that individuals resident in houses covered with materials such as plaster and wallpaper received average annual doses less than those living in houses with walls covered with wood and plastic paint, (P = 0.05). Therefore, the use of wallpaper and plaster to cover the parts of residential buildings is recommended. In addition, a comparison the type of materials used in buildings presented a significant difference (P = 0. 05) in radon gas concentration levels between buildings, that were constructed with adobe and concrete. However, there were no significant differences between buildings constructed with concrete and brick material, and those that were built with adobe and brick buildings (31). In another study, results showed that houses constructed with adobe emitted radon gas more than buildings made of concrete , brick and plaster (P < 0.05), while plaster walls emitted the lowest levels of radon gas (P < 0.01) (25). According to other researchers, adobe has a highly porous structure, thus scattering high levels of radon gas.

## 5. Discussion

It should be noted that the most studies in Iran are in high background radiation areas, which it could be lead to overestimation of results presentation. On the other the only published articles were examined in this study also the number of investigated studies were limited, probably many studies are published and it is so far a study was part of gray literature.

In this paper, studies involving the evaluation of radon levels in Iran with an emphasis on public exposure rather than occupational were reviewed. Most of the reported was associated with measurement of radon in water supplies; especially in high background radiation areas, such as Ramsar, and also indoor places with health hazards. Consequently, the highest values of radon gas in the water resources were reported in the hot springs of Mahallat and a house in the Talesh Mahalleh area of Ramsar. Most of the studies have proposed methods to reduce radon levels in water supplies, prior to its consumption by humans. In many of the studies involving the measurement of radon in residential buildings, the calculated dose received by humans was higher than the recommended amount. As for studies involving assessment of radiation levels in buildings, construction materials such as granite stone and adobe are thus not recommended. However, it should be noted that the evaluation of radon levels carried out in Iran, took place in a period of less than one year, where seasonal changes were not reported.
